# Observational Study of QuantiFERON®-TB Gold In-Tube Assay in Tuberculosis Contacts in a Low Incidence Area

**DOI:** 10.1371/journal.pone.0043520

**Published:** 2012-08-24

**Authors:** Emmanuel Bergot, Eglantine Haustraete, Brigitte Malbruny, Romain Magnier, Marie-Anne Salaün, Gérard Zalcman

**Affiliations:** 1 Service de pneumologie, CHU de Caen, Caen, France; 2 UMR 1086, Cancers et Prévention, CHU de Caen, Caen, France; 3 Centre de Prévention des Maladies Infectieuses, Caen, France; 4 Service de microbiologie, CHU de Caen, Caen, France; Hopital Raymond Poincare - Universite Versailles St. Quentin, France

## Abstract

**Background:**

QuantiFERON®-TB Gold in-Tube (QFT) assay is a recently developed test to assess latent tuberculosis infection in contagious tuberculosis (TB) contact subjects.

To assess the QFT assay in recently exposed contacts of active tuberculosis patients in a French area with low TB incidence but high Bacille Calmette-Guerin coverage, and evaluate progression rates to TB disease.

**Methodology/Principal Findings:**

Between January 2007 and December 2009, 687 contacts of culture-confirmed tuberculosis cases underwent the QFT assay, with tuberculin skin test (TST) in 473, and a 34 months mean follow-up. Of 687 contacts, 148 were QFT positive, while 526 were negative and 13 indeterminate. QFT was positive in 35% of individuals with TST ≥10 mm, 47.5% with TST ≥15 mm or phlyctenular, but in 21% of cases in which two-step TST (M0 and M3) remained negative. Conversely, QFT was negative in 69% of cases with two-step TST showing conversion from negative to positive. All indeterminate QFT were associated with TST induration <10 mm in diameter. For 29 QFT-positive subjects, no chemoprophylaxis was given due to medical contraindications. Of the remaining 119 QFT-positive contacts, 97accepted chemoprophylaxis (81.5%), and 79 (81.4%) completed the treatment. Two contacts progressed to TB disease: one subject was QFT positive and had declined chemoprophylaxis, while the other one was QFT negative. QFT positive predictive value for progression to TB was 1.96% (1/51) with a 99.8% (525/526) negative predictive value.

**Conclusions/Significance:**

Our results confirm the safety of the QFT-based strategy for assessing the TB chemoprophylaxis indication, as only one contact developed TB disease out of 526 QFT-negative subjects.

## Introduction

To prevent progression to active tuberculosis (TB) disease, the early diagnosis and treatment of recent TB infections were shown to be efficacious measures for identified contact individuals, leading to a good control of the TB burden, in countries with low TB incidence [1], [2]. A tuberculin skin test (TST) has long been the only reference test for the diagnosis of recent TB infection [3]. However, TST interpretation may be difficult in patients vaccinated with the Bacille Calmette-Guerin (BCG) vaccine [4], as the tuberculin protein shares common antigenic epitopes with the native *Mycobacterium tuberculosis* bacillus and BCG vaccine strain. Since the early 2000s, new assays have developed to detect *in vitro* gamma interferon production in response to specific *Mycobacterium tuberculosis* antigens (ESAT-6, CFP-10, TB7.7), which are not produced by the BCG strain. These interferon-gamma release assays (IGRAs) seemed to be a good alternative to the unspecific TST for the diagnosis of latent tuberculosis infection (LTBI) [5]. Indeed, the lack of a gold standard for the diagnosis of LTBI led to perform studies which compared TST and IGRA, particularly in tuberculosis contacts. In these studies, the concordance between IGRAs and TST was low, especially for the subgroup of contact individuals who previously received the BCG vaccine [6]–[8]. However, a strategy based on IGRA results still needs to be evaluated in terms of reducing the number of prophylaxis treatments administered compared to a TST-based strategy and in terms of the progression rates to active TB disease for both IGRA positive and negative groups. Only two published studies treat specifically the issue of contacts and use of IGRA in low incidence TB areas, but their diverging results led to conflicting interpretations as to the interest of IGRAs compared with TST for predicting the progression risk to active TB in the case of a positive assay. Both studies nonetheless confirmed the low risk of active TB in the case of negative IGRA [9], [10]. However, these studies included a limited number of contact subjects, 324 and 1033, respectively, with varying vaccination rates (81% and 52% BCG-vaccinated individuals, respectively), in 100% and 40% of migrants, respectively [9], [10], with such discrepancies justifying the discordant findings. Additional studies were deemed necessary to reassess the advantages of an IGRA-based strategy, which could eventually replace TST, since IGRA was shown to exhibit a better cost/benefit ratio in two recent pharmaco-economic studies [11], [12]. Our study aimed to evaluate QuantiFERON®-TB Gold In-Tube assay in individuals who were recently exposed to index cases with contagious pulmonary TB in Basse-Normandie, an area with low TB incidence (incidence of 7.3 cases per 100,000 inhabitants) but high BCG vaccine coverage (>80%) [13] and a low proportion of migrants (<5%) from high incidence countries. We report adhesion, compliance to chemoprophylaxis, and progression rates to active TB disease for both IGRA non treated-positive and negative groups. Finally, we also compared QFT and TST results in the subgroup of contact subjects having both procedures.

## Materials and Methods

### Study population

Between 1^st^ January 2007 and 31^st^ December 2009, this observational, prospective study enrolled all contacts of consecutive index cases of culture-confirmed pulmonary TB patients. Contact subjects were tested using QFT in the regional Centre for Infectious Disease Prevention (*Centre de Prévention des Maladies Infectieuses*, CPMI) in Caen, France. As part of the systematic investigation initiated after the diagnosis of a pulmonary TB case, the CPMI research nurses collected social and clinical data from contact subjects, and retrospectively estimated their contact time with the index case individual. If the cumulated contact time with the index case over the 3 months preceding the TB diagnosis was <8 hours, the contact was defined as “occasional”, if between 8 and 40 hours, “regular”, and if >40 hours, “close” [14]. As part of the screening procedure for recent TB infection in contact subjects, an initial evaluation (M0) comprising functional symptom assessment and chest X-ray with or without TST was proposed to each subject with regular or close contact. Additionally, the same evaluation procedure was proposed to 82 (11.9%) subjects with only occasional contact on account of their underlying immuno-compromised disease (n = 7) or because the index case was a health-care professional (n = 75) (Table 1). A second evaluation was performed 3 months later (M3), comprising a complete medical examination, chest X-ray, QFT and new TST, especially if the first TST revealed skin induration <10 mm in diameter, with QFT always performed prior to TST in such cases. Patients with a final diagnosis of active tuberculosis at M0 or M3 were excluded of the longitudinal study.

**Table 1:** Characteristics of close contacts undergoing QuantiFERON-TB® Gold in-tube assay.

Characteristics	QuantiFERON-TB® Gold in-Tube M3			
	Total	QFT positive	QFT negative	QFT indeterminate
	n (%)	n (%)	n (%)	n (%)
**Close contacts population**	687 (100)	148 (21.5)	526 (76.6)	13 (1.9)
**Age (years)**	44.5±18	47.5±17,3	42.6±17,9	0 (0)
**0–14**	5 (0.7)	0 (0)	5 (1)	0 (0)
**15–30**	149 (21.7)	23 (15.5)	126 (24)	0 (0)
**30–49**	312 (45.4)	61 (41.2)	251(47.7)	0 (0)
**50–64**	126 (18.3)	40 (27)	86 (16.3)	13 (100)
**≥65**	94 (13.7)	24 (16.2)	57 (10.8)	0 (0)
**Unknown**	1 (0.1)	0 (0)	1 (0.2)	0 (0)
**Sex**				
**Male**	208 (30.3)	62 (41.9)	144 (27.4)	2 (15.4)
**Female**	479 (69.7)	86 (58.1)	382 (72.6)	11 (84.6)
**French origin**				
**Yes**	660 (96.1)	138 (93.2)	509 (96.8)	13 (100)
**No**	26 (3.8)	10 (6.8)	16 (3)	0 (0)
**Unknown**	1 (0.1)		1 (0.2)	
**Immune suppression**				
**Yes**	6 (0,9)	1 (0.7)	5(1)	0 (0)
**HIV**	0 (0)	0 (0)	0 (0)	0 (0)
**Corticosteroids**	4 (0.6)	1 (0.7)	4 (0.8)	0 (0)
**Other treatment**	2 (0.3	0 (0	2 (0.4)	0 (0)
**BCG vaccination**				
**Confirmed**	140 (20.4)	28 (18.9)	112 (21.3)	0 (0)
**Unknown**	547 (79.6)	120 (81.1)	414 (78.7)	13 (100)
**Contact**				
**Household or intimate**	135 (19.7)	40 (27)	83 (15.8)	12 (92.3)
**Professional**	457 (66.5)	76 (51.4)	280 (53.2)	1 (7.7)
**Health care worker**	240 (34.9)	30 (20.3)	209 (39.7)	1 (7.7)
**Other**	95 (13.8)	32 (21.6)	63 (12)	0 (0)
**Exposure time**				
**Occasional**	82 (11.9)	16 (10.8)	65 (12.4)	1 (7.7)
**Regular**	368 (53.6)	76 (51.4)	292 (55.5)	0 (0)
**Intimate**	236 (34.4)	56 (37.8)	168 (31.9)	12 (92.3)
**Unkown**	1 (0,1)	0 (0)	1 (0.2)	0 (0)

The management strategy of contacts without evidence for active tuberculosis at M3 was based on the QFT results according to the 2006 French Health Authority recommendations [15].

Contact subjects with positive QFT were considered to have recent tuberculosis infection, and after full information, had the choice between a 3-month chemoprophylactic treatment with isoniazid and rifampicin, or 9-month chemoprophylactic treatment with isoniazid alone, or a clinical and radiological follow-up every 6 months for 2 years.

A clinical examination and chest x-ray were systematically proposed to every contact subject after 24 months of follow-up, including those with initial negative or indeterminate QFT.

Patients were informed of the need to seek medical help in case of functional or physical symptoms of active TB. At the end of the 24-month follow-up, contact subjects who failed to comply with the CPMI follow-up visits were contacted by telephone to confirm the absence of active TB symptoms. For subjects who died during follow-up, primary care physicians were asked to identify the cause of death and whether TB disease had been diagnosed. The regional health authority (DRASS), which collects compulsory TB declaration certificates from physicians or microbiology laboratories in the Calvados area, transmitted data to CPMI, thereby permitting a passive follow-up of contact subjects during the 24-month period and beyond.

### Tuberculin skin rest and QuantiFERON®-TB Gold in-Tube

TST was performed with an intradermal 0.1 ml injection of tuberculin equivalent to 5 IU Tubertest® in the front side of forearm, with the induration diameter being measured after 48–72 hours.

QFT was performed according to manufacturer's instructions (Cellestis Ltd, Chadstone, Australia) in the microbiology laboratory of Caen University Hospital. QFT results were expressed as positive or negative, using the cut-off value of ≥0.35 international units (IU)/mL.

QFT was considered as indeterminate if *i)* either an unprovoked IFN-γ level of ≥8.0 IU/mL in the negative-control plasma or *ii)* a IFN-γ ≤0.5 IU/mL on phytohemagglutinin stimulation with a level of IFN-γ in the tuberculosis antigen-exposed sample minus the level in the negative control of either <0.35 IU/mL or <25% of the IFN-γ concentration in the negative control plasma supplied by the manufacturer.

### Incident cases of tuberculosis disease

To define the positive or negative predictive values (PPV or NPV) of QFT, only cases of TB that occurred in contact subjects after the second clinical evaluation were considered to be “incident” and taken into account for evaluating the risk of progression towards TB.

Diagnosis presumption was based on clinical examination, chest x-ray or computed tomography scan, early-morning sputum, early-morning gastric aspiration (for subjects unable to spit), bronchoscopy with bronchoalveolar lavage (BAL) fluid specimens for acid-fast bacilli (AFB) smear examinations, Lowenstein-Jensen mycobacterial cultures, or histo-pathological data from tissue biopsies. Isolated contaminating strains from the index and incident cases were sent to the National Reference Centre for Mycobacteria and Resistance to Anti-tuberculosis Antibiotics (CHU Pitié-Salpêtrière, Paris) for molecular genotyping using the mycobacterial interspersed repetitive-unit (MIRU) typing to verify whether the strains were identical.

### Ethical considerations

The DRASS validated the CPMI annual report. All participants received written information describing the prospective screening strategy, which followed the national HAS recommendations [15]. They were informed that they were free to attend the follow-up medical visits or not, respond to telephone calls from CPMI nurses or not, and decline the scheduled x-chest exams. Clinical assessment was blinded as to the contact subject identity.

### Statistics

Concordance between TST and QFT results was assessed using κ coefficients for contacts. Kappa values <0.4 indicated weak correlation, values between 0.41–0.60 good agreement, and values >0.6 strong agreement.

QFT positive and negative predictive values (PPV and NPV) were calculated. QFT PPV for progression to tuberculosis is defined as the proportion of QFT positive untreated contacts who developed active tuberculosis during the follow-up. QFT NPV is defined as the proportion of QFT negative untreated contacts who do not progress to an active tuberculosis disease.

## Results

### Study population

**Figure 1:**
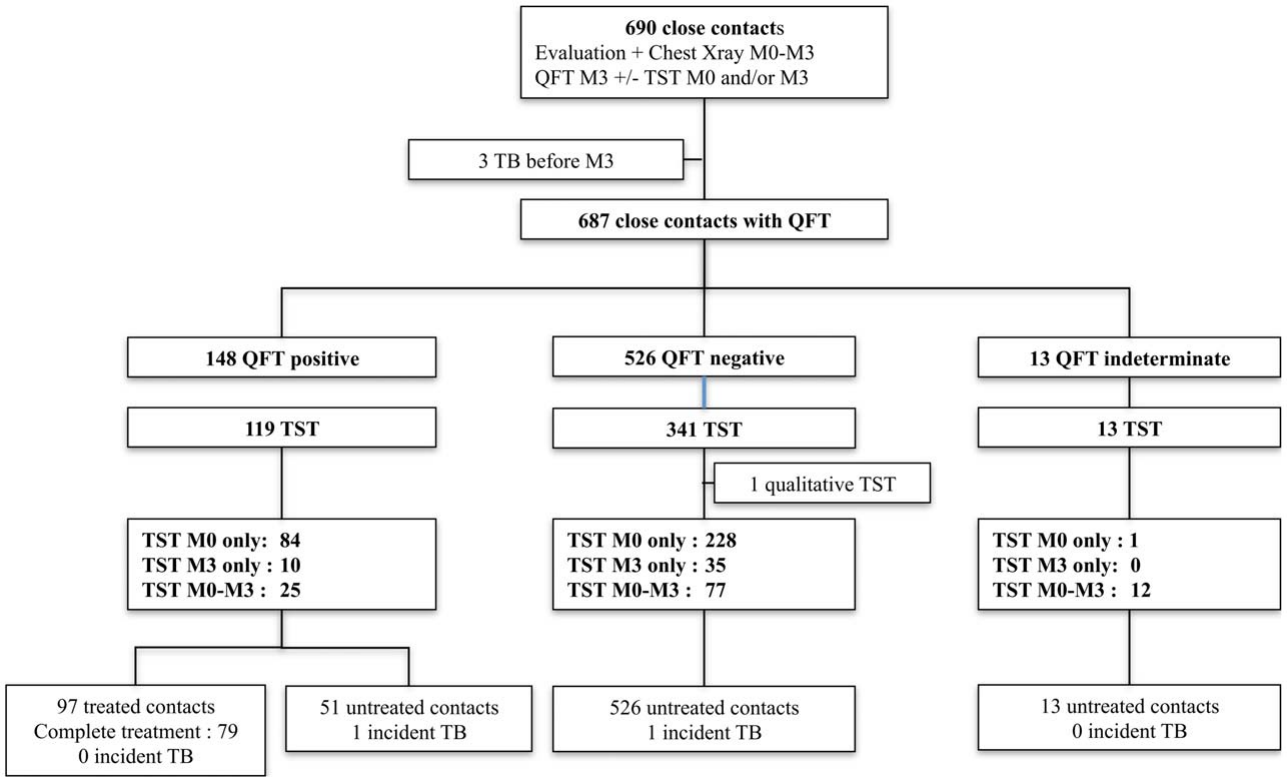
Flow chart of the study population. QFT: QuantiFERON®-TB Gold in-tube; TST: tuberculin skin test; M: month.

176 compulsory declaration certificates were received by the DRASS from the whole Basse-Normandie region, between 1^st^ January 2007 and 31^st^ December 2009. 690 contact individuals, corresponding to 46 index cases, were referred to CPMI, since living in Caen area and were enrolled in the current study. All 46 index cases of active TB disease were confirmed by positive Mycobacterium tuberculosis culture, with 82% having a positive AFB smear. Only one isolated strain was found to be resistant to isoniazid. Three cases of active TB were diagnosed at the second evaluation (M3) following chest X-ray, and excluded from the study. Those three subjects were close contacts, and had positive M3 QFT. Thus, 687 individuals were included in the final analysis (Figure 1). Contact subject characteristics are summarised in Table 1.

Median age was 42 years (11.5–97 years), with five contact subjects being younger than 15 years and 94 older than 65 years. The population subset aged 30–49 years was the most represented (312/687, 45.4%).Overall, 479 women and 208 men were enrolled. BCG vaccination status was proven by national or international vaccination certificates in only 140 subjects (20.4%). BCG scars were not systematically looked for. This low rate contrasts with the well-known BCG vaccine coverage in France, estimated to exceed 80%, since from 1950 until 19^th^ July2007, BCG vaccination was mandatory for children starting school at the age of 6, meaning that it was given to almost 100% of French people aged between 4 and 61 years.

Among the 687 contacts, 135 (19.7%) originated in the household or were related to the place of residency, while 457 (66.5%) derived from the occupational environment, with 240 (34.9%) pertaining to a medical or social profession. Only 26 contact subjects (3.8%) were born outside of France. None were HIV positive, while only seven (1%) received an immunosuppressive treatment for chronic disease.

Taking into account the estimated time of contact, there were 236 close contacts (34.4%), 369 (53.7%) regular, and only 82 (11.9%) occasional.

### QuantiFERON®-TB Gold in-Tube and TST

Among the 687 QFT performed at M3, 148 (21.5%) were considered as positive and 526 (76.6%) as negative, while 13 (1.9%) had indeterminate results. Population characteristics according to the QFT result are summarised in Table 1.

TST was conducted in 473 contact individuals: 313 at M0 alone, 45 at M3 alone, 114 at both M0 and M3. One contact with TST qualitative result was excluded from the comparative study(Figure 1). In contacts, the French Conseil Supérieur d'Hygiène Publique recommendations(3) defined tuberculosis infection as probable if skin induration was ≥10 mm. The tuberculosis infection was considered as “probably recent” if skin induration was ≥15 mm, or showed phlyctenular feature, or if the difference between an initial TST and a second TST at M3 was ≥10 mm in a two-step strategy. The tuberculosis infection was considered as only “possible” if skin induration was comprised between10 and 15 mm. Thus, to compare the QFT results with those different TST cut-offs, individuals who only had a TST at M0 with induration <10 mm in diameter(91 subjects), were excluded from the study.. For individuals for whom TST are available at both M0 and M3, the skin induration diameter at M3 was used for this comparison. Consequently, 381 TST results were taken into account: 222 individuals with TST only at M0 with skin induration ≥10 mm in diameter, 45 individuals with TST only at M3, and 114 with TST performed at both M0 and M3 (one contact subject was excluded due to only a qualitative result at M0 and M3) (Figure 1).

**Figure 2:**
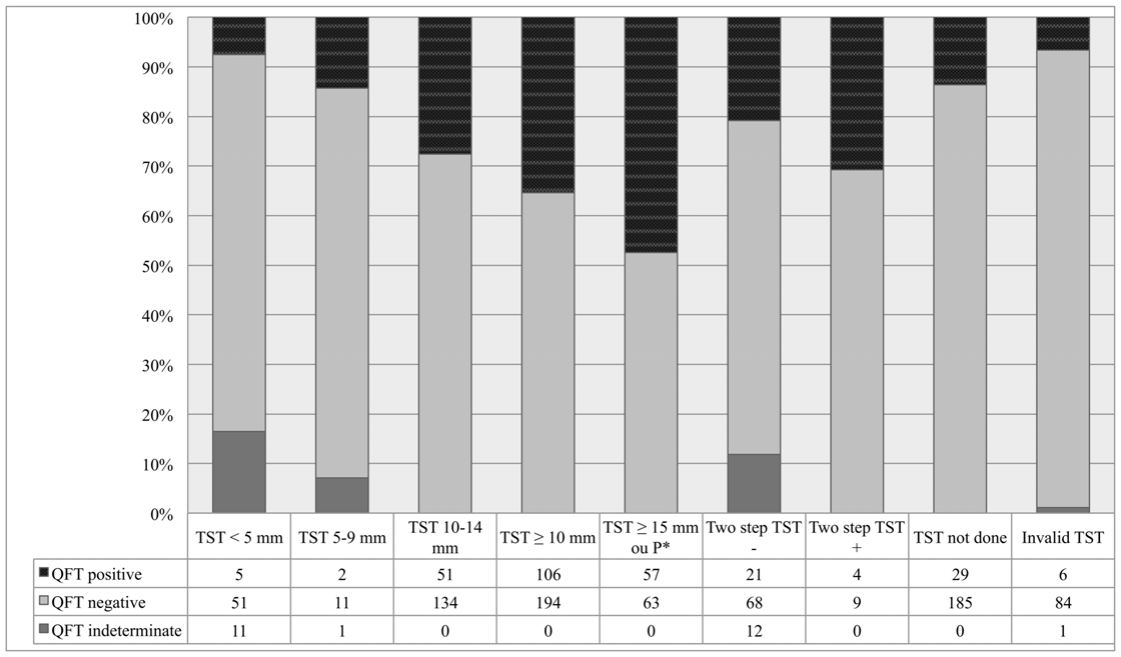
Concordance of QuantiFERON-TB® Gold in-tube (QFT) per tuberculin skin test (TST). QFT: QuantiFERON®-TB Gold in-tube; TST: tuberculin skin test; P: phlyctenular; M: month. *Three patients with 10–14 mm and phlyctenular TST.

QFT results expressed as a function of TST are provided in Figure 2. QFT was positive in 35% (106/300) of TST ≥10 mm and 47.5% (57/120) of TST ≥15 mm or phlyctenular.

Moreover, QFT was negative in 69% of cases with two-step TST being positive. Conversely QFT was positive in 21% of cases (21/101) in which two-step TST was negative.

All indeterminate QFT were associated with skin induration <10 mm in diameter. The concordance between M3 TST and M3 QFT was weak whatever the cut-off for positive TST (10 mm, 15 mm, or positive reaction) and whatever the subset according to age (Table 2 and 3).

**Table 2:** Agreement between QuantiFERON-TB® Gold in tube and tuberculin skin tests, analysed by BCG vaccination status, age, and health care worker status.

		QFT		Agreement		QFT		Agreement
	TST (≥10 mm)	Positive	Negative		TST (≥15 mm or P.)	Positive	Negative	
**Total**	Positive	28	50	Agreement = 61%	Positive	19	19	Agreement = 76%
**N = 147**	Negative	7	62	κ = 0.249; 95% CI (0.115; 0.382)	Negative	16	93	κ = 0.362; 95% CI (0.201;0.524)
**≥65 years**	Positive	10	4	Agreement = 85%	Positive	5	1	Agreement = 82%
**N = 62**	Negative	5	43	κ = 0.595; 95% CI (0.346; 0.844)	Negative	10	46	κ = 0.392; 95% CI (0.176;0.608)
**<65 years**	Positive	18	46	Agreement = 44%	Positive	14	18	Agreement = 72%
**N = 85**	Negative	2	19	κ = 0.109; 95% CI (−0.013; 0.232)	Negative	6	47	κ = 0.350; 95% CI (0.149;0.551)

TST: tuberculin skin test; QFT: QuantiFERON®-TB Gold in-tube; BCG: BacilleCalmette-Guérin.

**Table 3:** Agreement between QuantiFERON-TB® Gold in tube and two-step tuberculin skin tests, analysed by BCG vaccination status, age, and health care worker status.

		QFT		
	Two step TST^1^	Positive	Negative	Agreement
**Total**	Positive	4	9	Agreement = 71%
**N = 102**	Negative	21	68	κ = 0.051; 95% CI (−0.128;0.231)
**≥65 years**	Positive	1	2	Agreement = 73%
**N = 62**	Negative	14	45	κ = 0.033; 95% CI (−0.138;0.204)
**<65 years**	Positive	3	7	Agreement = 65%
**N = 40**	Negative	7	23	κ = 0.067; 95% CI (−0.243;0.377)

TST: tuberculin skin test; QFT: QuantiFERON®-TB Gold in-tube; BCG: Bacille Calmette-Guérin.

### Contact management according to QuantiFERON®-TB Gold in-Tube results and active TB incidence

According to the 2006 HAS recommendations authorising the use of QFT instead of TST for screening contacts of an index TB case [15], the management strategy for such individuals was based on the qualitative QFT results. Antibiotic chemoprophylaxis was only proposed to contact subjects with positive QFT regardless their TST results.

Among the 148 contact subjects with positive QFT, 97 (65.5%) were treated with the isoniazid plus rifampicin doublet (95%) or isoniazid alone (5%), whereas 51 subjects did not receive treatment on account of refusal in 22 cases or medical decision in 29. The treatment adhesion rate was 81.5%(97/119). Treatment compliance and tolerance were good for 79 out of the 97 (81.4%) individuals who received a full course of chemoprophylaxis. Chemoprophylaxis was interrupted due to adverse events in 10 cases (56%) or spontaneously in eight (44%) owing to their personal decision. No individuals with negative QFT received chemoprophylactic therapy.

Mean follow-up for the 687 contact subjects was 34±11 months, with two individuals presenting active TB during follow-up, as confirmed by mycobacterial culture. Molecular characterisation revealed both contacts to be infected by the same *M. tuberculosis* strain as their index case (Table 4). One of the patients had positive QFT and initial TST <10 mm in diameter at M0, but refused chemoprophylaxis. The other patient had negative QFT and TST induration of 13 mm at M0. Therefore, the rate of progression (PPV) towards active TB in contact individuals with positive QFT and who did not receive TB prophylaxis was estimated at 1.96% (1/51).Conversely, only one contact among 526 individuals with negative QFT subsequently developed active TB disease, providing a NPV of 99.8%. No active TB case was observed in contact subjects with undetermined QFT (Table 5).

**Table 4:** Characteristics of the two contacts with incident tuberculosis disease.

Case	Sex	Age, yrs	Origin	BCG status	ID	Type of contact	TST M0/M3, mm	QFT M3 (UI.ml^−1^)	CP	Time to TB, months	Type of TB	Culture	Strain identical to index case
**1**	M	34	France	UK	No	Intimate (Household)	5–9/ND	Positive (10)	Refusal	17	P	Positive	Yes
**2**	F	23	France	UK	No	Intimate	13/ND	Negative (0.12)	No	18	P	Positive	Yes

BCG: BacilleCalmette-Guérin; UK: Unknown; ID: immunodepression; TST: Tuberculin skin test; ND: not done; QFT: QuantiFERON®-TB Gold in-Tube; CP:Chemoprophylaxis; TB: Tuberculosis; P : Pulmonary.

**Table 5:** Number of cases of incident tuberculosis disease in untreated contacts.

QFT	Contacts		Progression to tuberculosis disease			
	n	Untreated, n	n	Sp,% (95% CI)	PPV, % (95% CI)	NPV, % (95% CI)
**Positive**	148	51	1	87 (63.9; 100)	1.96 (0; 4.2)	99.8 (99.4; 100)
**Negative**	526	526	1			
**Indeterminate**	13	13	0	NA	NA	NA

QFT: QuantiFERON®-TB Gold in-tube,;Sp: Specificity; PPV: Positive predictive value; NPV: Negative predictive value.

## Discussion

In this cohort study including recently exposed contacts of culture-proven tuberculosis patients from a low-incidence tuberculosis area, our results confirmthe interest of QFT in the diagnosis strategy of recent tuberculosis infection. Indeed, the high NPVfor subsequent development of TB disease (99,8%) showed the accuracy of IGRA as recently shown intwo meta-analyses [16], [17]. As in those meta-analyses, our studyalso showed a low PPV for IGRA. However a direct comparison between the current study and those papers can hardly be drawn, since the latest includedstudies with individuals having very various risks of evolution toward active tuberculosis (contact subjects, immunocompromised patients,adolescents, patients with chronic renal insufficiency…), and from areas of low to high tuberculosis incidence. Only three published works using a commercial IGRA have evaluated therisk of progression of contacts to active TB in a low incidence country [9], [10], [18]. Nevertheless,in the study by Bradshaw and al. [15], the infectiousness of the index case and the time of contact was not precisely described.

Thus, in our study, 21.6% of individuals had positive QFT. Several prior studies focused on similar populations living in low TB incidence areas. Our results reflect the findings of Diel *et al.* [10] (20.8% of positive QFT), while differing from those published by Kik or Bradshaw who respectively reported 52.5% and 8.6% rates of contact subjects with positive QFT [9], [18]. However, the discrepancy with the study by Kik *et al.* is probably due to the higher proportion of migrant individuals from high TB incidence countries (100% of subjects), compared with our study, in which only 3.8% of subjects were born in high-TB endemic areas, such as North or sub-Saharan Africa [9]. Hence, the proportion of positive QFT found in their study was related to previous TB infections [6]. For contact subjects with positive QFT, the adhesion rate to chemoprophylaxis was particularly high (97/119, 81.5%), which was superior to previous rates of 57% and 83.4% that relied only on TST for management [19], [20] as well as the 21.6% rate previously reported in a QFT-based study [10]. One limitation of our study was its mono-centre recruitment, as the most medical consultation was performed by the same chest physician, who was convinced of the relevance of QFT compared with TST in a population with high vaccine coverage. However, this may also account for our good adhesion results. The treatment completion rate was good (81.4%, with 18 treatment interruptions out of 97), being higher than previous data based on 9-month isoniazid monotherapy (52.6% to 64%) [21]–[24]. The use of 3-month doublet therapy for 95% of contacts could further improve this observance rate, being very close to the data reported by Martinez *et al.* [20].

For the 51 individuals with positive QFT who declined TB prophylaxis, the progression rate to active TB disease was only 1.96%. This result differs markedly from published rates, probably being a consequence of our study population characteristics. In Diel's study [10], 19 out of 147 contact subjects with positive QFT who did not receive treatment subsequently developed active TB, thus representing a progression rate of 12.9%. In this particular study, the progression risk to active TB was associated with age, with 28.6% of children versus 10.3% of adults (p = 0.03) developing TB. In our study, the low proportion of children under 15 (0.7%) may account for the difference in progression rates, with the five children included in the study all having negative QFT. In Diel's study [10], interferon-γ levels were also associated with the risk of progression to active TB (OR 1.93 for each unit of interferon-γ.ml^−1^, p<0.0001), with 16 of the 19 TB disease cases observed during follow-up having concentrations >3 UI.ml^−1^, with the remaining three <1 UI.ml^−1^ [10].

In our study, this difference could not be explained by a larger proportion of contact subjects having low interferon-γ concentrations, since for untreated contacts with positive QFT, interferon-γ levels <1 UI.ml^−1^ were only observed for 13, levels between 1 and 3 UI.ml^−1^ for 12, while levels >3 UI.ml^−1^ were found for 25. The duration of follow-up may have biased our study, since the mean follow-up period was 34 months in our study versus 46.6 months in that of Diel *et al.* However, all TB cases reported by Diel *et al.* occurred prior to the 23^rd^ month of follow-up [10]. In the Kik's study [9], the progression rate was 2.8% (5/178) and only involved adult migrants. Originating from a high TB incidence area is not only an independent risk factor for active TB [25], but also a risk factor for positive QFT, being related to past infection and leading to an over- or underestimation of the progression risk to active TB. The low proportion of migrants (3.8%) in our study makes this bias unlikely and therefore, our evaluation of QFT PPV may be considered reliable. However, we were not able to reliably evaluate the protective role of the BCG vaccine despite the high vaccination coverage in our region, since our methodology requiring a vaccination certificate to prove BCG history was obviously biased, underestimating the true rate of BCG coverage in our study population. Nevertheless, given the differences in BCG vaccination coverage as observed by Diel *et al.* (51.9%) [10], Kik *et al.* (80.8%) [10], and the estimated rate of 80% in France [13], the impact of these differences should be evaluated, as 74% of TB cases in Diel's study occurred in non-vaccinated patients [10]. None of 37 contacts with QFT positive progressed to TB in the Bradshaw's study [18].

After the begining of the current study, many publications reported a possible impact of a preceding TST on subsequent IGRA results. However those procedures were not performed in the context of a contact or outbreak study. A recent review, including thirteen studies, raised conflicting issues since five concluded to the absence of boosting of interferon-γ levels and seven to its occurrence [26]. In the studies reporting the occurrence of boosting, the risk was higher when IGRA was made shortly after TST (seven days). This risk persists up to 3 months after TST and wanes after this time. Boosting incidence was also higher in subjects who had a positive QFT before TST [26] whereas conversions only occurred in 2 to 12% ofinitially QFT-negative subjects [26]. In the latter group, subjects would receive inappropriate chemoprophylaxis on the basis of this falsely positive IGRA. In two recent studies, conversion persisted at day 84 or week 6, but occured only in subjects with a positive TST (≥10 mm) [27], [28]. These false positives could induce a bias in our study, and might explain our low PPV. Indeed, among the 148 M3 QFT-positive contacts, 119 had a TST at M0. 96 had a TST with an induration ≥10 mm. Thus, according to this risk, we could derive from our data that the maximum number of false positive cases in this seriescouldbe 2 to 13 (2 to 12% of 96) among the QFT-positive population. However, we feel this risk is problably overestimated, since IGRA was performedat least three months after TST. Moreover the most positive TST, especially phlyctenular or ≥15 mm induration, corresponded to subjects with recent tuberculosis infection and would have shown positive QFT, if IGRA had been made at M0. Therefore we deeply think the impact of the TST at M0 on subsequent QFT postitivity is low in our study. However, whereas TST had limited influence on QFT conversion from negative to positive, in QFT-positive subjects, M0 TST could actually have altered the absolute interferon-γ levels in our study, impairing the use of those quantitative values for predicting the risk of active tuberculosis.

Our results reassessed the safety of the QFT-based strategy, since for negative QFT our high NPV (99.8%) was similar to previously published studies on QFT (98 to 100%) [9], [10], [18].

Only one out of 526 contact subjects with negative QFT who did not receive prophylaxis subsequently developed active TB disease during follow-up without any satisfactory explanation. In contrast with previously reported cases, QFT was performed in this patient according to the recommendations, namely more than 2 months after the infectious contact. Moreover, re-infection with the index case was unlikely given that the index case had negative smears and cultures for *M. tuberculosis* at the time of hospital discharge.

Compared with a TST-based strategy, in our study, QFT may decrease the indications for TB chemoprophylaxis by 65% or 52% depending on the induration cut-off of 10 mm or 15 mm, respectively, without any consequence on the progression risk towards active TB. Our data confirms the findings of two pharmaco-economic studies [11], [12], including a French study in which the QFT-based strategy proved more efficacious with a better cost-benefit ratio.

### Conclusions

Our results confirm the advantage of QFT for the strategic management of TB contact subjects in a low TB incidence area with high BCG vaccination coverage and low rate of migrants or immuno-compromised individuals. However, larger studies are still required to confirm the advantage of QFT in identifying contact subjects with a higher risk of developing active TB according to population characteristics, such as vaccination coverage and geographic origin, and define the best management strategy for cases with undetermined QFT results.

## References

[1] American Thoracic Society : Targeted tuberculin testing and treatment of latent tuberculosis infection. Am J Respir Crit Care Med 161: s221–247. 10764341 10.1164/ajrccm.161.supplement_3.ats600

[2] Erkens CG, Kamphorst M, Abubakar I, Bothamley GH, Chemtob D, et al (2010) Tuberculosis contact investigation in low prevalence countries: a European consensus. Eur Respir J 36: 925–949. 20889463 10.1183/09031936.00201609

[3] [Intradermal reaction to tuberculin (IDR) or tuberculin test]. Rev Mal Respir 20: S27–33. 15101327

[4] Wang L, Turner MO, Elwood RK, Schulzer M, FitzGerald JM (2002) A meta-analysis of the effect of Bacille Calmette Guerin vaccination on tuberculin skin test measurements. Thorax 57: 804–809. 12200526 10.1136/thorax.57.9.804 PMC1746436

[5] Diel R, Goletti D, Ferrara G, Bothamley G, Cirillo D, et al (2011) Interferon-gamma release assays for the diagnosis of latent Mycobacterium tuberculosis infection: a systematic review and meta-analysis. Eur Respir J 37: 88–99. 21030451 10.1183/09031936.00115110

[6] Kik SV, Franken WP, Arend SM, Mensen M, Cobelens FG, et al (2009) Interferon-gamma release assays in immigrant contacts and effect of remote exposure to Mycobacterium tuberculosis. Int J Tuberc Lung Dis 13: 820–828. 19555530

[7] Arend SM, Thijsen SF, Leyten EM, Bouwman JJ, Franken WP, et al (2007) Comparison of two interferon-gamma assays and tuberculin skin test for tracing tuberculosis contacts. Am J Respir Crit Care Med 175: 618–627. 17170386 10.1164/rccm.200608-1099OC

[8] Diel R, Loddenkemper R, Meywald-Walter K, Niemann S, Nienhaus A (2008) Predictive value of a whole blood IFN-gamma assay for the development of active tuberculosis disease after recent infection with Mycobacterium tuberculosis. Am J Respir Crit Care Med 177: 1164–1170. 18276940 10.1164/rccm.200711-1613OC

[9] Kik SV, Franken WP, Mensen M, Cobelens FG, Kamphorst M, et al (2010) Predictive value for progression to tuberculosis by IGRA and TST in immigrant contacts. The European respiratory journal : official journal of the European Society for Clinical Respiratory Physiology 35: 1346–1353. 10.1183/09031936.00098509 19840963

[10] Diel R, Loddenkemper R, Niemann S, Meywald-Walter K, Nienhaus A (2011) Negative and Positive Predictive Value of a Whole-Blood Interferon-{gamma} Release Assay for Developing Active Tuberculosis: An Update. Am J Respir Crit Care Med 183: 88–95. 20802162 10.1164/rccm.201006-0974OC

[11] Diel R, Wrighton-Smith P, Zellweger JP (2007) Cost-effectiveness of interferon-gamma release assay testing for the treatment of latent tuberculosis. Eur Respir J 30: 321–332. 17504793 10.1183/09031936.00145906

[12] Deuffic-Burban S, Atsou K, Viget N, Melliez H, Bouvet E, et al (2010) Cost-effectiveness of QuantiFERON-TB test vs. tuberculin skin test in the diagnosis of latent tuberculosis infection. Int J Tuberc Lung Dis 14: 471–481. 20202306

[13] (2001) Mesure de la couverture vaccinale en France. Bilan des outils et des méthodes en l'an 2000. Institut de veille sanitaire. 1–57 p.

[14] Enquête autour d'un cas cas. Recommandations pratiques.

[15] [HAS evaluation: interferon gamma detection test for the diagnosis of tuberculous infections]. Med Mal Infect 37: 689–693. 18232083 10.1016/j.medmal.2007.05.008

[16] Diel R, Loddenkemper R, Nienhaus A (2012) Predictive value of interferon-gamma release assays and tuberculin skin testing for predicting progression from latent TB infection to disease state: a meta-analysis. Chest 10.1378/chest.11-3157 22490872

[17] Rangaka MX, Wilkinson KA, Glynn JR, Ling D, Menzies D, et al (2012) Predictive value of interferon-gamma release assays for incident active tuberculosis: a systematic review and meta-analysis. Lancet Infect Dis 12: 45–55. 21846592 10.1016/S1473-3099(11)70210-9 PMC3568693

[18] Bradshaw L, Davies E, Devine M, Flanagan P, Kelly P, et al (2011) The role of the interferon gamma release assay in assessing recent tuberculosis transmission in a hospital incident. PLoS One 6: e20770. 21695149 10.1371/journal.pone.0020770 PMC3113857

[19] Gershon AS, McGeer A, Bayoumi AM, Raboud J, Yang J (2004) Health care workers and the initiation of treatment for latent tuberculosis infection. Clin Infect Dis 39: 667–672. 15356781 10.1086/422995

[20] Martinez Alfaro E, Solera J, Serna E, Cuenca D, Castillejos ML, et al (1998) [Compliance, tolerance and effectiveness of a short chemoprophylaxis regimen for the treatment of tuberculosis]. Med Clin (Barc) 111: 401–404. 9834911

[21] LoBue PA, Moser KS (2003) Use of isoniazid for latent tuberculosis infection in a public health clinic. Am J Respir Crit Care Med 168: 443–447. 12746255 10.1164/rccm.200303-390OC

[22] Menzies D, Dion MJ, Rabinovitch B, Mannix S, Brassard P, et al (2004) Treatment completion and costs of a randomized trial of rifampin for 4 months versus isoniazid for 9 months. Am J Respir Crit Care Med 170: 445–449. 15172892 10.1164/rccm.200404-478OC

[23] Menzies D, Long R, Trajman A, Dion MJ, Yang J, et al (2008) Adverse events with 4 months of rifampin therapy or 9 months of isoniazid therapy for latent tuberculosis infection: a randomized trial. Ann Intern Med 149: 689–697. 19017587 10.7326/0003-4819-149-10-200811180-00003

[24] Page KR, Sifakis F, Montes de Oca R, Cronin WA, Doherty MC, et al (2006) Improved adherence and less toxicity with rifampin vs isoniazid for treatment of latent tuberculosis: a retrospective study. Arch Intern Med 166: 1863–1870. 17000943 10.1001/archinte.166.17.1863

[25] Figoni J AD, Che D (2011) Tuberculosis cases notified in France. Bulletin Epidemiologique Hebdomadaire 22: 258–260.

[26] van Zyl-Smit RN, Zwerling A, Dheda K, Pai M (2009) Within-subject variability of interferon-g assay results for tuberculosis and boosting effect of tuberculin skin testing: a systematic review. PLoS One 4: e8517. 20041113 10.1371/journal.pone.0008517 PMC2795193

[27] van Zyl-Smit RN, Pai M, Peprah K, Meldau R, Kieck J, et al (2009) Within-subject variability and boosting of T-cell interferon-gamma responses after tuberculin skin testing. Am J Respir Crit Care Med 180: 49–58. 19342414 10.1164/rccm.200811-1704OC

[28] Sauzullo I, Massetti AP, Mengoni F, Rossi R, Lichtner M, et al (2011) Influence of previous tuberculin skin test on serial IFN-gamma release assays. Tuberculosis (Edinb) 91: 322–326. 21664872 10.1016/j.tube.2011.05.004

